# The Multimorbidity Questionnaire (MMQ1): English translation and validation of a Danish patient reported outcome measure for quality of life in people with multiple long-term conditions in a cross-sectional survey

**DOI:** 10.1007/s11136-025-03901-6

**Published:** 2025-01-23

**Authors:** Kieran Sweeney, Kristine Bissenbakker, Volkert Siersma, Alexandra Jønsson, Eddie Donaghy, David Henderson, Stewart W. Mercer, John Brandt Brodersen

**Affiliations:** 1 Usher Institute, University of Edinburgh, Edinburgh, UK; 2 Centre of General Practice, Department of Public Health, Faculty of Health Sciences, University of Copenhagen, Copenhagen, Denmark; 3 Research Unit for General Practice, Region Zealand, Denmark; 4 Research Unit for General Practice, Department of Community Medicine, Faculty of Health Sciences, UiT The Arctic University of Norway, Tromsø, Norway; 5 Section of Health and Society, Department of People and Technology, Roskilde University, Roskilde, Denmark

**Keywords:** Multimorbidity, Multiple long-term conditions, Quality of life, Patient reported outcome measures

## Abstract

**Purpose:**

MMQ1 is a Danish-language patient-reported outcome measure (PROM) for quality of life (QOL) in people with multiple long-term conditions (MLTC). It measures needs-based QOL across six scales: Physical ability, Concerns and worries, Limitations in daily life, Social life, Personal finances and Self-image. There is currently no such measure available in English. This study aimed to translate and validate MMQ1 for use in the United Kingdom.

**Methods:**

Translation used a two-panel method (expert panel: n = 5; and lay panel: n = 6). Content validity was assessed via cognitive interviews (n = 6). A postal survey of 2,753 patients with MLTC recruited through eight GP practices in Scotland included EQ-5D-5L and ICE-CAP as comparator measures alongside MMQ1. Classical test theory psychometric analysis of survey responses followed the International Society for Quality of Life Research minimum reporting standards.

**Results:**

Translation resulted in an English-language MMQ1 with good face validity. Cognitive interviews established good content validity. 597 survey responses were received (response rate 22%). Good internal consistency reliability and concurrent validity were demonstrated. Confirmatory factor analysis showed acceptable fit to the six-scales of MMQ1. Five of the six scales also demonstrated good discriminative ability for detecting clinically meaningful differences in QOL. However, inter-item and inter-scale correlations suggested item redundancy and scale overlap.

**Conclusion:**

The English-language MMQ1 demonstrated adequate psychometric properties using classical test theory. Further validation using Rasch analysis is planned, and may help optimise and abbreviate the measure. This PROM has the potential to improve the measurement of QOL in MLTC research, including trials.

**Supplementary Information:**

The online version contains supplementary material available at 10.1007/s11136-025-03901-6.

## Introduction

The growing number of people living with multiple long-term conditions (MLTC, multimorbidity) is one of the most significant challenges facing healthcare systems globally. In the UK, more than one in three adults live with MLTC, and in older people and those living in more deprived areas, it is now the norm [1–3]. People with MLTC are likely to experience poorer health outcomes and lower quality of life (QOL). Healthcare services – which are largely orientated around single conditions – face growing pressure from the increased demand and higher costs associated with MLTC [4–6].

In light of this challenge, there has been rapid growth in MLTC research over the past two decades, including several trials of interventions to improve outcomes for people with MLTC. However, there remains no clear evidence of benefit from these interventions [7]. Whether this represents intervention failure or problems with the outcome measures used is unclear.

Quality of life (QOL) is often chosen as the primary outcome for trials because it is an intuitive, holistic concept that is important to patients and influences healthcare utilisation [8]. While single-disease trials often use disease-specific patient reported outcome measures (PROMs) to measure QOL, there are currently no QOL PROMs that are designed specifically for people with MLTC. Consequently, MLTC trials have relied on generic measures such as EQ-5D-5L and SF-36 [7, 9–11]. These generic PROMs are considered broadly applicable and have well-established validity in many contexts [12]. However, their development often predates the widespread use of modern psychometric techniques known as modern test theory^1^ and they have certain well-recognised problems. EQ-5D-5L, for example, exhibits limited sensitivity to change [13, 14], and is prone to differential item functioning in heterogenous populations (that is, unwanted variations in the psychometric performance of items between demographic groups) [15]. The limitations of using generic PROMs in MLTC research in particular have been highlighted previously [16, 17].

The development of bespoke outcome measures for people with MLTC has therefore been identified as a research priority [8, 17–19]. Although a number of MLTC-specific PROMs have been developed in recent years, these measure concepts such as treatment burden and illness perception rather than QOL per se, so are less suitable as primary outcome measures in trials. Moreover, all of these MLTC-specific PROMs exhibited risk of bias according to the COSMIN checklist (which assesses the quality of PROM design and validation according to modern test theory) in a recent systematic review [17].

### Development of the Multimorbidity Questionnaire (MMQ)

In response to this, the Multimorbidity Questionnaire (MMQ) – a Danish-language PROM for people with MLTC – was recently developed and validated using Modern Test Theory methods at the University of Copenhagen. It’s qualitative groundwork, conceptualisation and development have been described elsewhere [20–22]. It has two parts: MMQ1, measuring needs-based QOL; and MMQ2, measuring self-perceived inequity. These were conceived as independent but coherent constructs, designed to be used either separately or together [23]. The present paper focusses only on the translation and validation of MMQ1 given its explicit focus on QOL. The translation and validation of MMQ2 will be reported separately.

MMQ1, measuring needs-based QOL, is made up of 37 items across six domains: physical ability, self-determination, security, social life, self-image and personal finances. Needs-based QOL is a well-established concept that is widely used in disease-specific QOL PROMs [24], and is based on Maslow’s Hierarchy of Needs and the assumption that life gains quality from a person’s ability to achieve their goals and fulfil their needs [25]. As such, it is a more patient-centred concept than health-related QOL (as measured by EQ-5D-5L), and has also been shown to be more responsive [26]. In the Danish validation study, MMQ1 demonstrated strong psychometric properties using Rasch analysis [22]. It has since been used as an outcome measure in a large-scale trial of a new model of care for people with MLTC in Denmark (the MM600 trial) [27]. The six domains of MMQ1 were developed as independent scales, resulting in six separate scores rather than a single sum score.

### Aim

The aim of this study was to translate and validate MMQ1 for use in the UK.

## Methods

### Phase 1: Translation, adaptation and content validation

The translation of MMQ1 from Danish to English was conducted using a two-panel method [28]. The first panel took place in Copenhagen in January 2023. It consisted of three native English speakers living in Denmark overseen by members of the research team (KB and JBB) who were also bilingual. This group produced a preliminary ‘expert’ translation of MMQ. The second panel took place in Edinburgh in March 2023 and consisted of a focus group with six members of a Patient and Public Involvement (PPI) network established to support research within the Advanced Care Research Centre (ACRC) at the University of Edinburgh. Participants had diverse socioeconomic backgrounds and levels of education, and all had experience of living with long term conditions, however, as a PPI group rather than formally recruited research participants, consent was not obtained for recording or reporting characteristics. The focus group was also attended by the Danish research team (KB, JBB, ABRJ) in order to maintain consistency with the original meaning of the items, and was facilitated by a member of the Edinburgh research team (KS). The process involved using a *think-aloud* approach to review the preliminary translation line-by-line, with *verbal probing* used to explore any semantic ambiguity and improve the translation for a lay audience [29, 30]. Changes were made to the translation if there was agreement between lay participants that suggested changes improved clarity, and the Danish research team felt that the original meaning was preserved. Formal, subsequent back translation was not performed, although the bilingual fluency of the Danish team negated the need for this, as translation accuracy could be checked in real-time. In addition to refining the translation, participants’ impressions of how understandable, appropriate and relevant the measure seemed (its face validity) were also discussed.

The resulting translation was then piloted in six cognitive interviews taking place in August and September 2023, with patients purposively sampled from two GP practices in Lothian, one in an urban area of high deprivation and one in a rural area of mixed deprivation. Suitable patients identified by GPs were those living with MLTC where the GP felt that the health conditions were likely to be affecting QOL. Sampling was also targeted such that there was a balance of genders and a weighting towards participants from more deprived areas and/or with experience of mental-physical multimorbidity. This weighting was chosen as it was hypothesized that impact on QOL might be greater in these groups, and therefore questionnaire items and responses reflecting this could be suitably piloted. Nine suitable patients were identified by their GP, either opportunistically or based on clinical familiarity, and contacted by their GP by telephone. All nine agreed to be contacted by the research team with further information. Of these, six responded to contact from the research team and agreed to proceed to interview. Cognitive interviewing uses qualitative techniques (including think-aloud and verbal probing) on a one-to-one basis to assess how a respondent processes each item, thereby reducing measurement error [29, 30]. Six interviews were conducted in two rounds, with some initial analysis and modification taking place after the first four interviews, prior to minor amendments and further testing. Verbal probing explored the importance, relevance and clarity of each item, concept coverage (content validity) and responder burden. Interviews were recorded and transcribed, and field notes documented.

### Phase 2: Psychometric validation in a survey

The translated questionnaire was then included in a survey of 597 adults recruited from eight GP practices in Lothian. Full details of the sampling process including search criteria is included in the Supplementary File. In summary, practices were recruited by volunteering in response to an advert circulated by NHS Research Scotland Primary Care Network. In the eight practices that were recruited, practice lists were searched electronically for adult patients on two or more chronic disease registers, or on four or more repeat medications (a method used previously by SWM to identify patients with MLTC [31]). Patients were excluded if they had already been recruited to another research study by the Primary Care Network. Using these criteria, 11,860 potentially eligible patients were identified across eight practices. From these, a random sample of 2,800 patients was selected for screening. The size of the sample screening list in each of the eight practices ranged from 200 to 400 (mean 350) and was determined by the discretion of the participating GP. The sampling lists were screened to remove any patients deemed inappropriate for survey inclusion (such as those with dementia or approaching end-of-life). From 2,800 patients (across eight practices) included after random sampling of eligible patients, 2,753 patients were retained for invitation to complete the survey (47 deemed unsuitable by their GP). From the 2,753 distributed surveys, 597 responses were received (22%). This higher than expected response rate exceeded the target of 400 responses, chosen for consistency with the Danish MMQ validation study [22]. Surveys were posted in November and December 2023 and collection stopped at the end of January 2024. No reminders were sent, and no reimbursement provided.

Survey packs included a cover letter, a participant information sheet, the questionnaire and a pre-paid return envelope. The questionnaire itself (see Appendix 1) included the translated MMQ1 and MMQ2; two demographic items (age and gender); a checklist of 17 common chronic conditions (to assess multimorbidity) [32]; a bespoke single-item global QOL rating (“*I would say my overall quality of life is: very good / good / acceptable / poor / very poor*”); a bespoke feedback item to assess responder burden (“*Thinking about the questions answered so far, I would say the questionnaire felt: far too long / too long / about right / too short / far too short”)*; two comparator QOL measures (EQ-5D-5L, ICE-CAP) to assess concurrent validity with MMQ1 [33, 34], and one comparator measure for MMQ2 (CARE measure) [35].

### Analysis

Data were analysed using SPSS version 27 and R version 4.2.2. Practices were grouped according to whether they served mainly deprived, mixed, or affluent populations. Psychometric analysis of MMQ1 using classical test theory was guided by the International Society for Quality of Life Research (ISOQOL) minimum reporting standards [36], as well as the COSMIN Risk of Bias checklist [37], as detailed below. Analyses for each scale were based on complete responses for that scale. Responses with a missing value in a given domain were excluded from analyses of that scale. As such, the number of complete responses included for analysis varied slightly for each scale.

### Conceptual framework

Described elsewhere [20] and summarised above.

### Translation

Two-panel method described above.

### Face and content validity

The importance, relevance, clarity and coverage of items was initially explored in the focus group, and then in greater depth during cognitive interviews (see Phase 1 above). Cognitive interview transcripts were thematically analysed using a framework approach [38].

### Scale properties

Completion rates, and floor-effects (proportion of responses scoring zero) were compared to the comparator measures (EQ-5D-5L, ICE-CAP).

### Dimensionality (structural validity)

Confirmatory factor analysis (CFA) was performed on R using the *lavaan* package, with a diagonally weighted least squares estimation method given the ordinal nature of the data, and an independent clustering model. Six separate CFA models were fitted to the six scales in MMQ1, given that these were developed to be used independently, generating six separate scores. Factor loadings and overall measures of fit (Comparative Fit Index (CFI), Root Mean Square Error of Approximation (RMSEA), and Standardized Root Mean Square Residual (SRMR)) were calculated. CFI > 0.95, RMSEA < 0.06 and SRMR < 0.08 indicate good fit [39].

### Concurrent validity

EQ-5D-5L and ICE-CAP are well-established QOL PROMs, for which high positive correlation with MMQ1 was hypothesized. Correlation was assessed using Spearman correlation coefficients (due to non-linearity of the variables) in a matrix including the six MMQ1 scale scores, the individual item scores from the comparator measure (five items in EQ-5D-5L, and five in ICE-CAP) and the sum scores for the comparator measures. The correlation matrix also included MMQ1 total scores (i.e., the sum of all six scale scores, even though these are technically independent scales), as well as the single-item global rating of QOL, in order to further examine concurrent validity. EQ-5D-5L may be considered a gold standard measure of quality of life (HRQOL), so this comparison assessed both construct and criterion validity as defined by ISOQOL.

### Convergent and discriminant validity

Multiscale analysis was also performed, calculating the correlation (Spearman coefficient) between items in a given scale and the remaining sum score for that scale (corrected item-total correlation) and comparing this with the correlation between the same items and the sum score for the other scales [40]. Items are expected to demonstrate higher correlation with their native scale (convergent validity) than other scales within the measure (discriminant validity). Inter-scale correlations were also examined, with high values (> 0.6) suggesting substantial overlap between domains.

### Reliability

Internal consistency was tested by way of inter-item correlation matrix, Cronbach’s alpha, and scale reliability with items dropped. Cronbach’s alpha values above 0.7 were interpreted as indicating good internal consistency reliability [39]. Scale reliability should be reduced rather than improved when items are dropped. Optimal inter-item correlation is between 0.20–0.40, with higher values suggesting homogeneity and redundancy of items within the scale [41].

### Responsiveness

This was not assessable as it requires repeat measurement. However, future work is planned to assess the performance of MMQ1 in longitudinal surveys.

### Interpretability of scores

Scores for MMQ1 are to be reported as six individual scale scores. In order to aid interpretation of these values in future studies and to assess the discriminative ability of each scale, the distribution of MMQ1 scale scores were plotted with data grouped according to responses to the single-item global QOL rating. The mean scale scores (plus SD) were compared for each global item response category (Very good, Good, Acceptable, Poor or Very poor). Discriminative ability is reported as the number of individuals needed in a t-test with 5% significance and 80% power to find a clinically meaningful difference between known groups. In this case this was taken as the difference between consecutive global item response categories (i.e., between very good/good, good/acceptable, acceptable/poor, poor/very poor). A low number of individuals (< 75) indicates a highly discriminative scale. The same analysis was performed in the Danish MMQ validation study, allowing for direct comparison.

### Responder burden

Responder views regarding the length of the questionnaire were explored during cognitive interviews and by way of a feedback item included in the survey (which used a 5-point Likert scale to assess agreement with the statement “*The questionnaire felt too long*”). Investigator burden was not assessed in this study.

## Results

### Phase 1: Translation, adaptation and content validation

#### Translation

The PPI focus group proposed several revisions to the wording of the preliminary ‘expert’ translation to enhance its clarity and accessibility for English-speaking respondents. For example, the item ‘*My illnesses prevent me from spontaneous activities’* in the preliminary translation was changed to ‘*My health conditions prevent me from doing things on the spur of the moment*’. Subsequently, two further minor wording changes were made following the first round of cognitive interviews (see Supplementary File), before the MMQ was piloted in two more interviews and finalised for the postal survey.

#### Face and content validity

Focus group participants, who were involved in the lay translation of the questionnaire, endorsed the relevance, importance and coverage of the six domains within MMQ1 as a reflection of needs-based quality of life. This was supported by the in-depth cognitive interviews (see quotes in Supplementary Figure 3). Table 1 shows the characteristics of each participant in the cognitive interviews.

**Table 1:** Characteristics of cognitive interview participants

Participant	Age group	Gender	Mental-physical multimorbidity	Psychosocial complexity*	SIMD	LTC
P1	60–70	Male	No	No	9	3
P2	40–50	Female	Yes	Yes	2	4
P3	70–80	Female	No	No	4	4
P4	60–70	Male	No	Yes	3	5
P5	70–80	Female	Yes	Yes	7	7
P6	50–60	Female	No	Yes	1	5

*LTC* Long term conditions (count), *SIMD* Scottish Index of Multiple Deprivation (decile 1 = most deprived)

*Psychosocial complexity included presence of addiction, social isolation, or employment/welfare/housing challenges

### Phase 2: psychometric validation in a survey

Table 2 shows the characteristic of the 597 survey respondents. The mean age of respondents was 70 years (range 21–97 years) and 48% were male. The proportion of practices serving populations with mixed levels of deprivation was 58%, while 28% served mainly deprived areas, and 14% affluent. The median number for long term conditions (LTCs) was 4 (range 0–11). 59 respondents (10%) self-reported fewer than two long term conditions. 28% of respondents had mental-physical multimorbidity compared with 62% physical only.

**Table 2:** Survey respondent characteristics (N = 597)

Characteristic	Frequency n (%)
Mean age (SD)	70 (13)
Age (years)	
< 50	41 (7)
51–60	75 (13)
61–70	155 (26)
71–80	190 (32)
81–90	117 (20)
> 90	14 (2)
Missing	5 (1)
Gender	
Male	285 (48)
Female	310 (52)
Missing	2 (0)
Socioeconomic group*	
Deprived	167 (28)
Mixed	347 (58)
Affluent	83 (14)
Number of LTCs	
0–1	59 (10)
2	117 (20)
3	119 (20)
4	123 (21)
5	74 (12)
6	52 (9)
7+	53 (9)
Multimorbidity type	
Mental-physical	165 (28)
Physical only	373 (62)
None**	59 (10)

*LTC* Long term conditions, *SD* standard deviation

*Practice-level deprivation

**Patients who self-reported 0–1 conditions

#### Scale properties

Completion rate (Table 3) for each of the six scales in MMQ1 ranged between 96 and 99%, meaning that only 1–4% of scale responses had missing data. Completion was lowest (96%) for scale three (Limitations in daily life) which has ten items, and scale one (Physical ability) which has six items. It was highest (98%) for scale five (Personal finances), which has three items. Similar completion rates were found for EQ-5D-5L and ICE-CAP (97%). Responses were positively skewed, with a substantial floor effect demonstrated in all six scales, ranging from 14% (scale two*)* to 61% (scale five, Personal finances). Across MMQ1 as a whole, it was 7%, compared with 12% for EQ-5D-5L and 16% in ICE-CAP.

**Table 3:** Scale properties

Scale title Item content		Scale floor effect: n/N (%)	Complete responses n (%)	CFA factor loadings (SE)	Cronbach’s alpha (Item dropped)
1. Physical ability		130/570 (23%)	570 (96%)		0.925
1a *I’m frustrated that I can do so little*			581 (97%)	0.906 (0.010)	(0.907)
1b *Physical activity for pleasure is out of the question*			585 (98%)	0.919 (0.010)	(0.907)
1c *My physical limitations prevent me from maintaining my personal hygiene without support*			582 (97%)	0.832 (0.022)	(0.934)
1d *My physical limitations prevent me from doing household chores, such as shopping, laundry, cleaning *			586 (98%)	0.914 (0.011)	(0.908)
1e *I have to push myself to keep going physically*			586 (98%)	0.918 (0.010)	(0.905)
1f *My health conditions keep me from being as active as I would like to be*			592 (99%)	0.950 (0.007)	(0.904)
2. Concerns and worries		78/575 (14%)	575 (96%)		0.918
2a *I worry about my health conditions*			586 (98%)	0.913 (0.010)	(0.898)
2b *I’m concerned about the treatment I receive for my health conditions*			581 (97%)	0.801 (0.020)	(0.915)
2c *My health conditions make me worry about the future*			585 (98%)	0.929 (0.009)	(0.895)
2d *I worry about my physical abilities*			583 (98%)	0.897 (0.012)	(0.896)
2e *I have to make an effort to stay mentally strong*			586 (98%)	0.833 (0.016)	(0.908)
2f *I worry that my health conditions burden my*			586 (98%)	0.829 (0.017)	(0.908)
3. Limitations in daily life		122/570 (21%)	570 (96%)		0.961
3a *My health conditions limit where I can go*			591 (99%)	0.934 (0.009)	(0.955)
3b *My health conditions prevent me from doing things on the spur of the moment*			584 (98%)	0.957 (0.006)	(0.954)
3c *I feel my health conditions trap me in my own home*			587 (98%)	0.938 (0.009)	(0.956)
3d *My health conditions affect what I can and can’t do*			588 (98%)	0.938 (0.009)	(0.956)
3e *When I have to do something different, I have to plan it well in advance*			586 (98%)	0.911 (0.010)	(0.957)
3f *My health conditions make it difficult to plan far in advance*			589 (99%)	0.909 (0.011)	(0.956)
3 g *Because of my health conditions, I feel dependent on others in everyday life (e.g. family, friends, neighbours, carers, home helps *etc*.)*			589 (99%)	0.919 (0.011)	(0.956)
3 h *I feel limited in everyday life because I lack the necessary support from the health and social care system*			587 (99%)	0.860 (0.019)	(0.962)
3i *Because of my health conditions, I have difficulty keeping my home the way I want*			590 (99%)	0.871 (0.015)	(0.958)
3j *Because of my health conditions, it is proving difficult to pursue my hobbies*			590 (99%)	0.889 (0.013)	(0.958)
4. My social life		270/574 (47%)	574 (96%)		0.910
4a *My health conditions make it difficult to spend time with family, friends and others*			582 (97%)	0.920 (0.012)	(0.886)
4b *I feel I’m a burden to others because of my health conditions*			582 (97%)	0.908 (0.013)	(0.887)
4c *In dealing with my health conditions, I feel a lack of support from my social network (e.g. family, friends, neighbours, carers, home helps *etc*.)*			584 (98%)	0.844 (0.024)	(0.906)
4d *I could do with a close relative or friend who has the time and energy to help me on a daily basis*			580 (97%)	0.868 (0.020)	(0.898)
4e *I feel my health conditions prevent me from establishing new relationships (e.g. friends, colleagues, dating *etc*.)*			584 (98%)	0.900 (0.016)	(0.891)
4f *Because of my health conditions, I find it hard to provide emotional support to my loved ones*			584 (98%)	0.868 (0.018)	(0.892)
5. Personal finances		355/582 (61%)	582 (98%)		0.909
5a *I feel my health conditions limit my ability to be financially stable*			582 (97%)	0.951 (0.011)	(0.850)
5b *I’m worried about my finances*			585 (98%)	0.957 (0.011)	(0.874)
5c *I’m worried about my social status because of unemployment, poor finances or similar*			584 (98%)	0.950 (0.011)	(0.884)
6. Self-image*		178/572 (31%)	572 (96%)		0.892
6a *I feel embarrassed about the impact and limitations my health conditions cause*			589 (99%)	0.939 (0.011)	(0.860)
6b *My health conditions lower my self-esteem*			585 (98%)	0.956 (0.010)	(0.857)
6c *It is unpleasant being seen as a patient by others*			582 (97%)	0.897 (0.015)	(0.868)
6d *I feel people judge me because of my health conditions*			583 (98%)	0.868 (0.018)	(0.872)
6e *I blame myself for my health conditions*			583 (98%)	0.657 (0.035)	(0.885)
6f *I often feel guilty about my lifestyle (e.g. smoking, alcohol, diet, exercise) in relation to my health conditions*			587 (98%)	0.558 (0.040)	(0.895)
EQ-5D-5L		68/579 (11%)	579 (97%)		
ICE-CAP		95/581 (16%)	581 (97%)		

*SE* standard error, *CFA* Confirmatory factor analysis

*CFA model for scale six (Self image) include a correlation term 6e ~~ 6f

#### Dimensionality

CFA factor loadings for the six scales in MMQ1 are shown in Table 3. Factor loadings ranged from 0.83 to 0.95 for six items in scale one; 0.80–0.93 for six items in scale two; 0.86–0.96 for ten items in scale three; 0.84–0.92 for six items in scale four; 0.95–0.96 for three items in scale five; and 0.75–0.95 for six items in scale six. The six CFA models showed an acceptable fit with the data using CFI, RMSEA and SRMR as fit indices (Table 4). For Physical ability (scale one), Limitations in daily life (scale three) and My social life (scale four), all three indices indicated a good fit (CFI > 0.95, RMSEA < 0.05, SRMR < 0.08). In the case of Concerns and worries (scale two), CFI and SRMR were good, while RMSEA was acceptable at 0.054. For Personal finances (scale five), the model was just-identified with only three items, making these fit measures not applicable. The CFA model for Self-image (scale six) initially showed poor fit (RMSEA 0.190, SRMR 0.105, CFI 0.989). Further analysis revealed high collinearity between items 6e and 6f, with a modification index of 183 (compared with less than 30 for other item pairs) suggesting possible local dependence between these conceptually similar items (“*I blame myself for my health conditions*” and “*I often feel guilty about my lifestyle in relation to my health conditions*”). Adding a correlation term between these two items (6e ~~ 6f) improved the model fit indices substantially (CFI 1.00, RMSEA 0.024, SRMR 0.018) while maintaining acceptable to good factor loadings (0.56–0.96), as detailed in Supplementary Table 4.

**Table 4:** Measure-of-fit indices for scale CFA models

Scale	CFI	RMSEA	SRMR
1. Physical ability	1.000	0.042	0.019
2. Concerns and worries	0.999	0.054	0.025
3. Limitations in daily life	1.000	0.032	0.020
4. My social life	1.000	0.013	0.017
5. Personal finances	n/a	n/a	n/a
6. Self-image (model 1)	0.989	0.190	0.105
*6. Self-image (model 2)	1.000	0.024	0.018

*CFI* Comparative Fit Index, *RMSEA* Root Mean Square Error of Approximation, *SRMR* Standardized Root Mean Square Residual

*Model 2 includes correlation term: 6e ~~ 6f

#### Concurrent validity

As hypothesized, MMQ1 demonstrated strong correlation with EQ-5D-5L and ICE-CAP. Spearman correlation coefficients (Supplementary Table 2) between the six scales of MMQ1 and the EQ-5D-5L total score ranged from 0.52 (Personal finances) to 0.85 (Physical ability), with an average of 0.73. The correlation between the MMQ1 total (i.e., the sum of the six scale scores) and EQ-5D-5L score was 0.86. For ICE-CAP, correlation with MMQ1 total was 0.81, and correlation for individual scales ranged from 0.56 (personal finances) to 0.77 (social life) with an average of 0.71. For the single-item global rating of QOL, correlation with MMQ1 total was 0.77, while scale correlations ranged from 0.51 (personal finances) to 0.73 (limitations in daily life), with an average of 0.67.

#### Convergent and discriminant validity

Corrected item-total correlations (Supplementary Table 3) between the 37 items and their native scales ranged from 0.60 to 0.90 (scale averages 0.71 to 0.82), indicating acceptable convergent validity. For each 37 items, corrected item-total correlations were higher for the native scale than any of the other five scale scores, indicating good discriminant validity. However, inter-scale correlation (Supplementary Table 5) ranged from 0.50 (between scales one and five) to 0.90 (between scales one [Physical ability] and scale three [Limitations in daily life]), with an average coefficient of 0.69. These high values, particularly for the correlation between scales one and three, suggest substantial overlap between the domains of MMQ1.

#### Reliability

As shown in Table 3, Cronbach’s alpha for the six scales ranged from 0.89 to 0.96, indicating very strong internal consistency reliability. Scale reliability was lower with items dropped for all 37 items except items 3 h and 6f. Inter-item correlation matrices for the six scales are shown in Supplementary Table 1. Average coefficients in each matrix (i.e., each scale) ranged from 0.58 (scale six, Self-image) to 0.78 (scale five, Personal finances). These values are above the optimal range, suggesting a degree of homogeneity and possible redundancy.

#### Interpretability

**Fig. 1:**
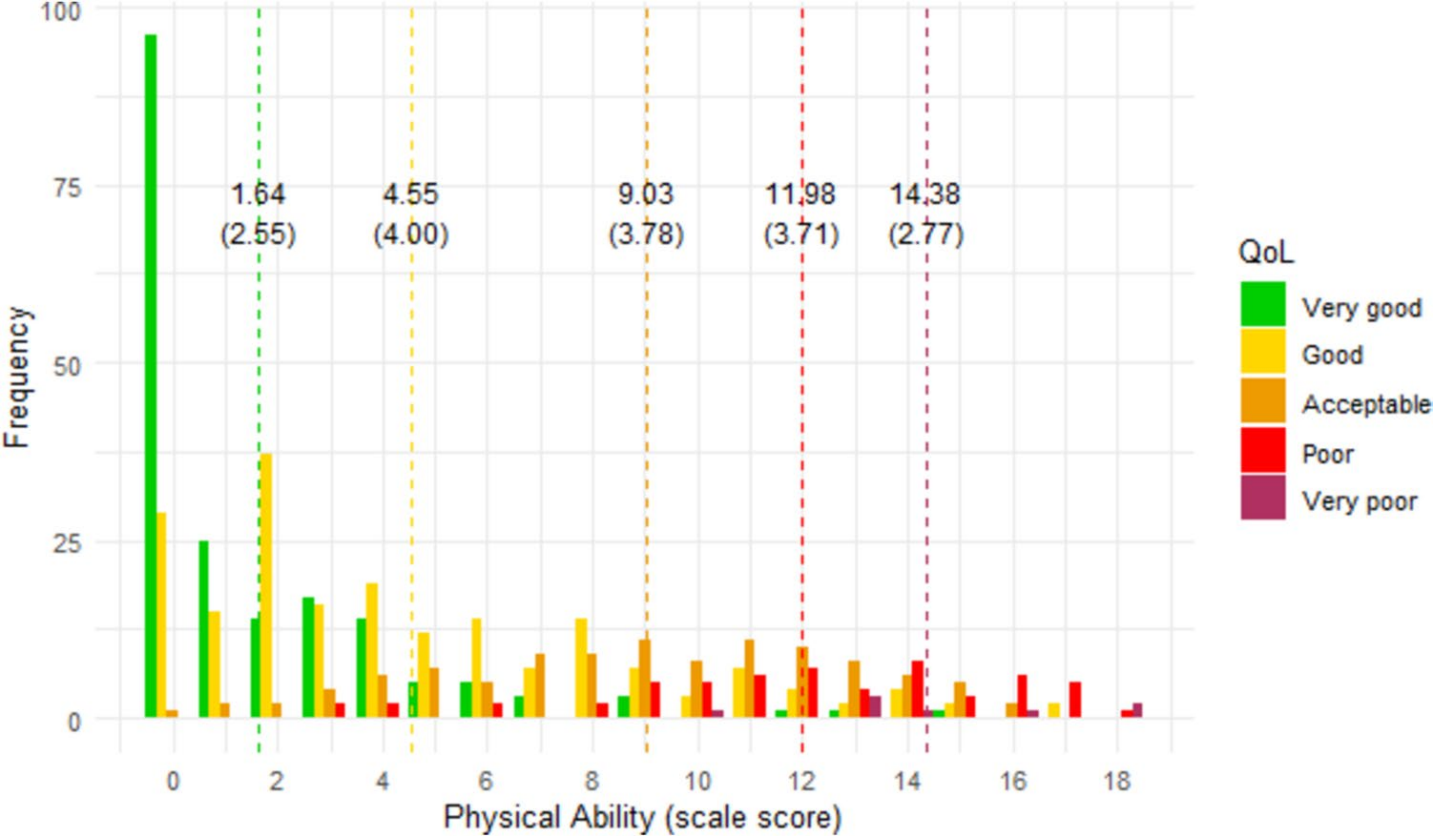
Distribution of ‘physical ability’ score by global QOL rating. Dotted vertical lines show the mean scale score (and standard deviation) for each global item response group

**Fig. 2:**
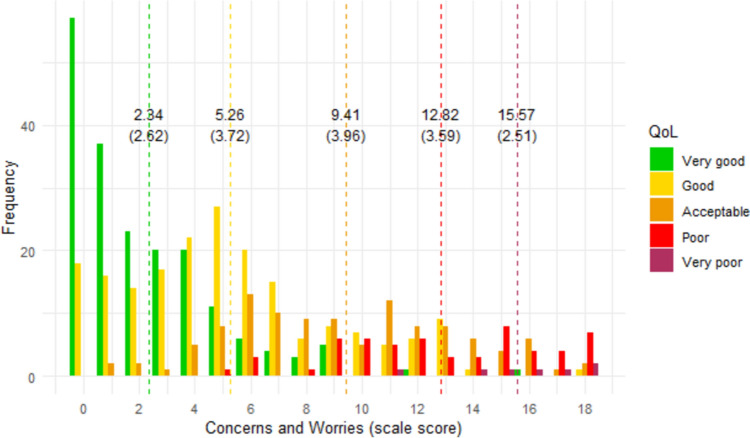
Distribution of ‘concerns and worries’ score by global QOL rating. Dotted vertical lines show the mean scale score (and standard deviation) for each global item response group

**Fig. 3:**
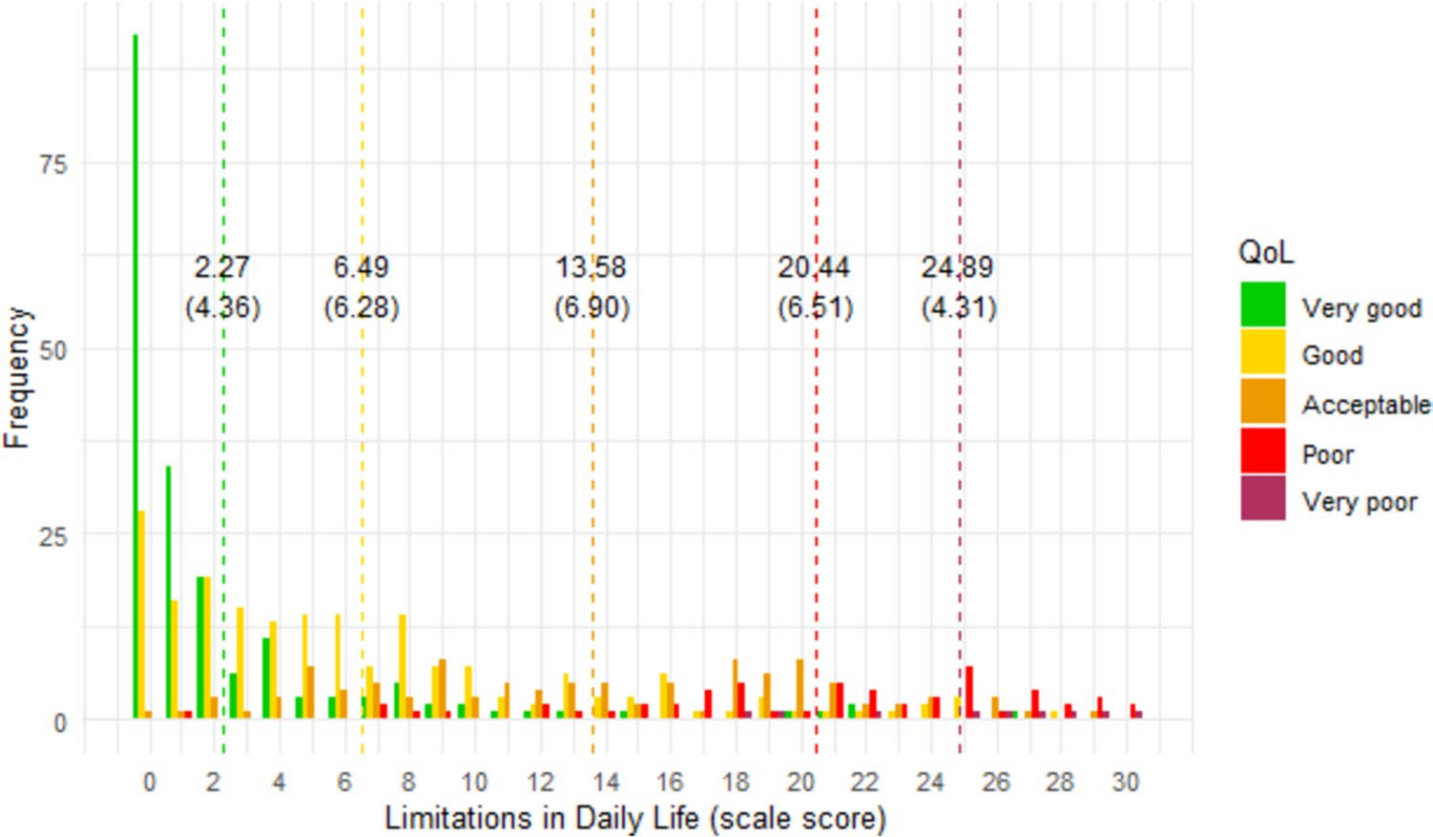
Distribution of ‘limitations in daily life’ score by global QOL rating. Dotted vertical lines show the mean scale score (and standard deviation) for each global item response group

**Fig. 4:**
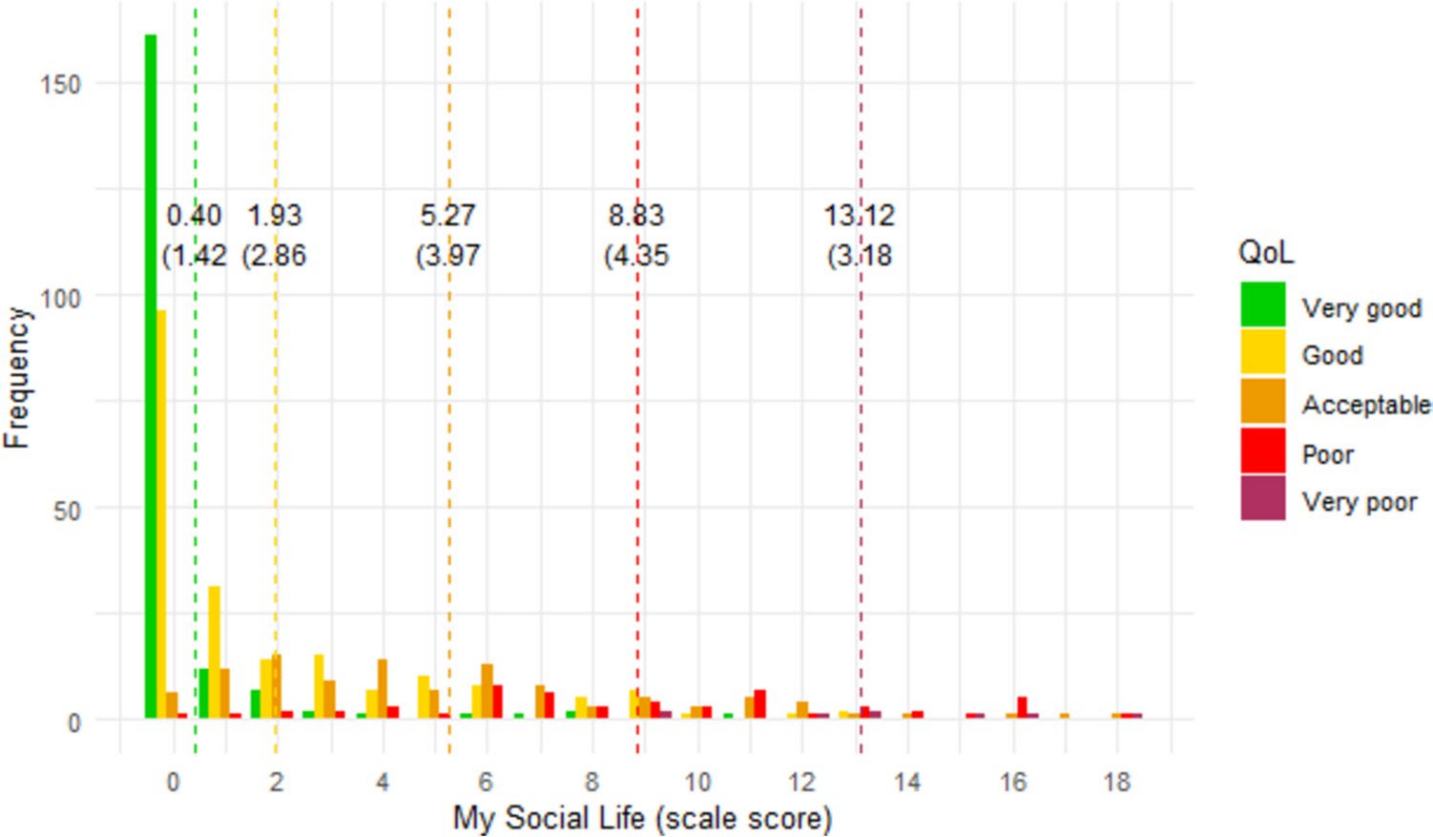
Distribution of ‘social life’ score by global QOL rating. Dotted vertical lines show the mean scale score (and standard deviation) for each global item response group

**Fig. 5:**
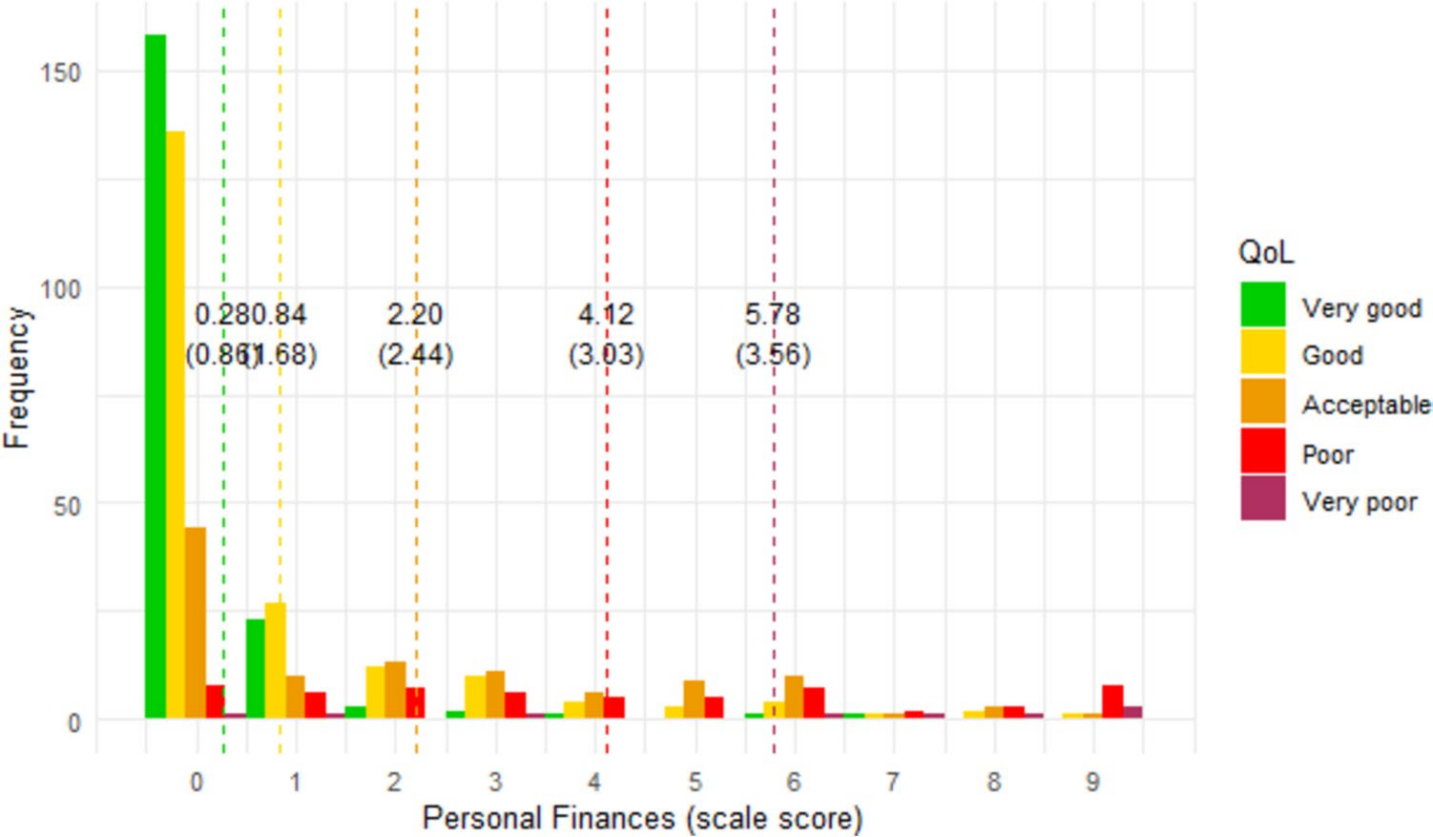
Distribution of ‘personal finances’ score by global QOL rating. Dotted vertical lines show the mean scale score (and standard deviation) for each global item response group

**Fig. 6:**
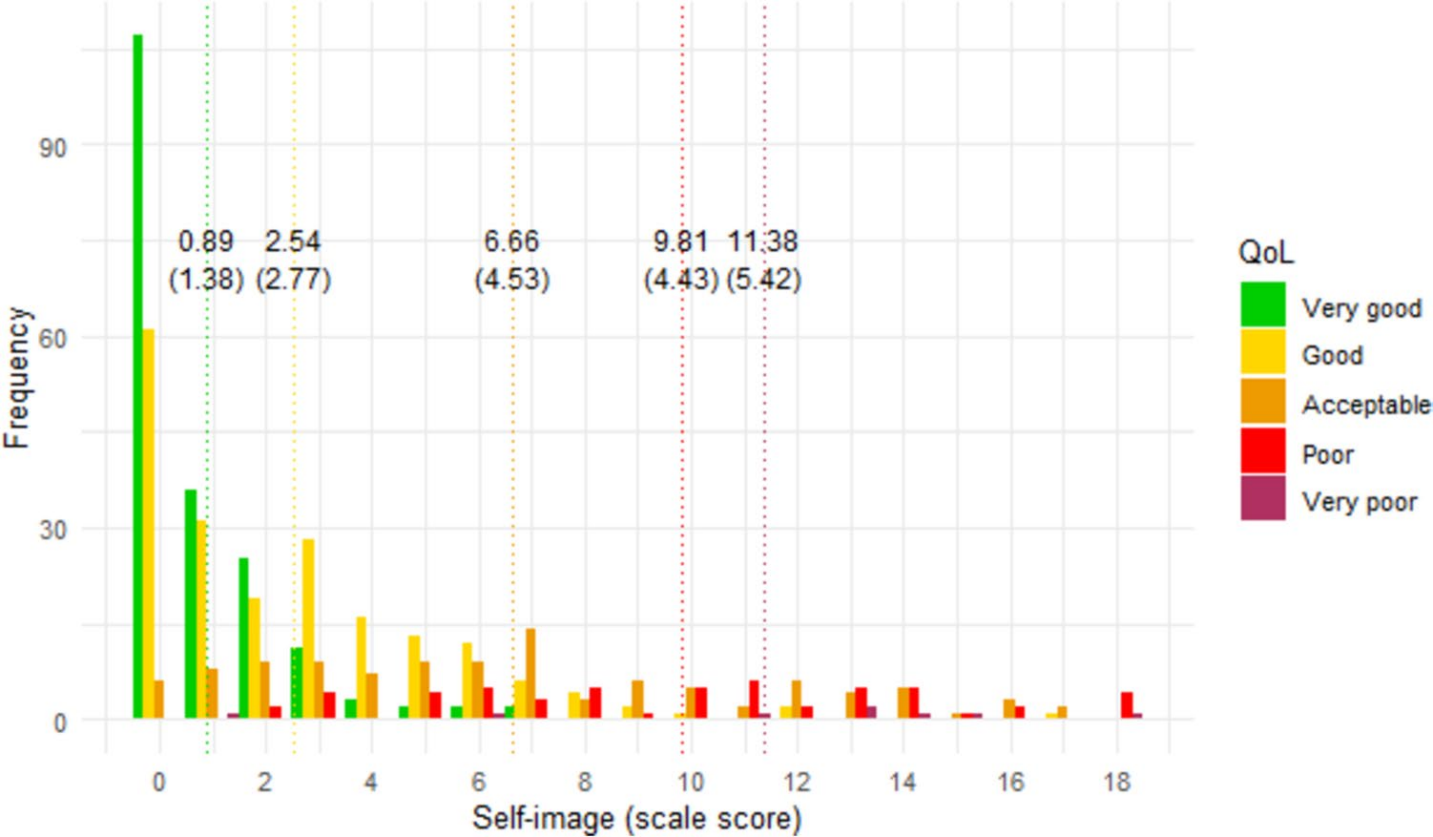
Distribution of ‘self-image’ score by global QOL rating. Dotted vertical lines show the mean scale score (and standard deviation) for each global item response group

Figures 1, 2, 3, 4, 5, 6 show the distribution of scale scores grouped by responses to the single-item global quality of life rating. Table 5 shows the discriminative ability of each scale – that is, the sample size required for a t-test with 5% significance and 80% power to discriminate between consecutive categories of the single-item global quality of life rating. As Table 4 shows, scale five (personal finances) was the least discriminating scale. For all other scales, a sample size of less than 75 (and in most cases less than 50) is needed to discriminate between individuals with a global quality of life rating of good-vs-acceptable, or acceptable-vs-poor.

**Table 5:** Discriminative ability of six MMQ1 scales

Scale	“Very good” to “Good”	“Good” to “Acceptable”	“Acceptable” to “Poor”	“Poor” to “Very poor”
1. Physical ability	62	28	54	78
2. Concerns and worries	54	32	46	56
3. Limitations in daily life	72	32	34	70
4. My social life	112	48	50	36
5. Personal finances	282	104	82	148
6. Self-image	92	40	68	382

This is the number of individuals required for a t-test with 5% significance and 80% power to discriminate between consecutive response groups in the global QoL item

#### Responder burden

Of the 534 respondents who completed the feedback item, only 13% did not feel the questionnaire was too long. In the earlier cognitive interviews, most participants also felt that the measure should be shorter (see quotes in Supplementary Figure 3).

Table 6 summarises the findings, and maps the analysis to the ISOQOL and COSMIN standards [36, 37].

**Table 6:** Summary of findings by ISOQOL and COSMIN standards

COSMIN standards	Comment	Outcome
PROM development	Conceptual development and item generation described previously	n/a
	Translation described	Satisfactory
Content validity	Assessed in cognitive interviews	Satisfactory
Structural validity	Assessed using confirmatory factor analysis	Satisfactory
	Inter-scale correlation also assessed	Indicates scale overlap
Internal consistency	Cronbach alpha assessed	Satisfactory
	Inter-item correlation also assessed	Indicates item redundancy
Measurement invariance	Differential item functioning not assessed	Further work required
Reliability (test–retest)	Not assessable in this study	Further work required
Measurement error	Not assessable in this study	Further work required
Criterion validity	Assessed using EQ5D	Satisfactory
Construct validity	Assessed using EQ5D and ICE-CAP	Satisfactory
Responsiveness	Not assessable in this study	Further work required

ISOQOL standards	Comment	Outcome
Conceptual and measurement model	Conceptual framework described previously	n/a
	Scale properties assessed	Floor effect noted
	Dimensionality assessed by confirmatory factor analysis	Satisfactory
	Inter-scale correlation assessed	Indicates scale overlap
Reliability	Cronbach’s alpha assessed	Satisfactory
	Inter-item correlation	Indicates item redundancy
Validity	Content assess in cognitive interviews	Satisfactory
	Construct/criterion validity assessed using EQ5D and ICE-CAP	Satisfactory
	Responsiveness not assessable in this study	Further work required
Interpretability	Informed using discriminative ability assessment	Satisfactory
Translation	Two-panel method described	Satisfactory
Respondent and investigator burden	Respondent burden assessed	Indicates need to abbreviate

## Discussion

In this study we have translated, adapted and validated a multi-scale PROM for needs-based QOL in people living in UK with MLTC. MMQ1 contains 37 items over 6 independent scales: Physical ability, Concerns and worries, Limitations in daily life, Social life, Personal finances and Self-image. These scales have a strong conceptual basis, and demonstrate acceptable content validity, internal consistency, structural validity, and concurrent validity. All of the scales, with the exception of Personal finances (which only contains three items), have strong discriminative ability for detecting clinically meaningful differences in QOL as measured by a single global item. However, a substantial floor effect was noted in all scales, and high inter-item and inter-scale correlations point to item redundancy and scale overlap.

The finding of high floor effects may, in part, be attributable to the sampling method and in particular how it differed from that of the Danish validation study. While the Danish study excluded patients who rated their health as “good” or “very good” [22], no such exclusion existed in the present study. Instead, we used an electronic sampling method to randomly identify adult patients with MLTC, possibly resulting in a “healthier” sample population than in the Danish study. This discrepancy highlights wider inconsistencies in the concept and definition of MLTC/multimorbidity and raises the question as to whether MMQ1 is more suitable for use in those with so-called “complex multimorbidity” [42]. The highest floor effect (61%) was seen in the Personal finances scale. This might partly be due to it being the shortest scale, with only three items, making a score of zero more likely than in longer scales. Additionally, this domain may reflect a higher severity of QOL impact than the other scales, in so far as limitations in daily life, or concerns and worries, are likely to arise before an individual’s personal finances are materially affected. Although the six scales of MMQ1 are not designed to produce a single sum score, they are intended to be used alongside each other, and so it is notable that only 7% of respondents scored zero in all six domains, which was lower than the floor effect found in EQ-5D-5L and ICE-CAP.

CFA demonstrated acceptable structural validity in the six scales of MMQ1, although potential local dependence between two items in scale six (Self-image) lead to the inclusion of a correlation term in the model for that scale. Although the pattern of item-total correlations (Supplementary Table 3) supported the scales’ discriminant and convergent validity, there were also high inter-item and inter-scale correlations (Supplementary Tables 1 and 5), suggesting item redundancy and overlap between domains. Respondent feedback also favoured shortening the measure. Taken together, these findings suggest that future work to refine and abbreviate MMQ1 may be of value. This may involve Rasch analysis of the study data, which would provide greater item-level detail, and, coupled with our understanding of the items’ content validity, help determine whether MMQ1 can be shortened without compromising its measurement properties. Future analysis may also involve testing for differential item functioning in relation to key variables such as age, sex, socioeconomic status and multimorbidity, something which is a recognised limitation of generic outcome measures in heterogenous populations such as MLTC.

The use of six independent scales in this PROM has both advantages and disadvantages, particularly in the context of clinical trials. Each scale addresses a different aspect of QOL, allowing for a more nuanced and multifaceted assessment of this broad concept, measurement of which is not well served by reducing it to a single score. This design has the potential to enhance sensitivity by detecting domain-specific changes that a single score might miss. It also offers flexibility, with the option to only use, or to prioritise, relevant scales according to a study’s objectives. On the other hand, this design also adds complexity for researchers in terms of score interpretation, statistical analysis, and power calculations. However, the analysis of discriminative ability (Table 5) shows that the required sample sizes to discriminate between Good/Acceptable or Acceptable/Poor in the global QOL item are less than 75 for five out of the six scales.

In general, the psychometric validation of PROMs on an aggregate, population level does not guarantee their reliability on an individual level, where greater fluctuation and error is expected [23, 43]. In keeping with this, MMQ1 was not designed for use in a clinical setting, but rather as a research tool for use in observational and intervention studies. Nevertheless, in practice PROMS are often used by clinicians to explore patients’ views and priorities, and facilitate discussions around treatment options [44]. Reflecting this, the original Danish MMQ1 has been used as both a communication aid and outcome measure in the pilot study of an intervention for people with severe mental illness and MLTC in Denmark (the SOFIA trial) [45].

### Strengths and limitations

This study used a robust approach to translation, adaptation and content validation, and involved a survey of a large sample size, using a battery of questionnaires: MMQ1, EQ-5D-5L and ICE-CAP, plus MMQ2 and CARE (not reported in this paper). The response rate was relatively low (22%) and representativeness could not be assessed, so the possibility of response bias could not be excluded. Future work involving comparison between sample and population characteristics will be required to explore this.

This psychometric validation study, in line with the recommended use of MMQ1, treated scale scores as missing if one or more of its items had missing values. Hence, validity was assessed for completers only. Including incomplete scale values in the present study would have necessitated strong assumptions and introduced potential distortion, while the potential gain in statistical power is minimal: completion rates for the 37 items are between 97 and 99% (1–3% missing), and completion rates for the six scales are between 96 and 99% (1–4% missing). When using MMQ1 as an outcome measure in future studies however, it will be necessary to impute missing values in order to avoid bias and preserve statistical power. Since missing values occur at the scale level, the validity established for completers implicitly extends to the non-completers.

Feedback pertaining to the length and response burden of MMQ1 should also be considered in the context of the overall response burden of the survey pack. EQ-5D-5L and ICE-CAP were used to assess concurrent validity, but it should be noted that these measure health-related QOL which is conceptually distinct from needs-based QOL as measured by MMQ1. Finally, there was also no opportunity to assess responsiveness, measurement error, or test–retest reliability as this study only involved a single cross-sectional survey. However, the high values of Cronbach’s alpha are important given that this is considered the lower limit for reliability [46].

## Conclusion

MMQ1 is a multi-scale PROM for needs-based QOL in people with MLTC, which has been translated and adapted from the original Danish, and validated in a UK setting. It demonstrated acceptable psychometric properties using classical test theory, albeit with evidence of item redundancy and scale overlap which, along with responder feedback, support the need to refine and shorten the measure, if psychometrically feasible. There also remains a need to assess responsiveness in a before-and-after survey. To our knowledge this is the first English-language measure of QOL that is bespoke for people with MLTC. As such, it has the potential to improve the measurement of QOL in MLTC intervention trials.

## Supplementary Information

Below is the link to the electronic supplementary material.

Supplementary file1 (DOCX 1160 kb)

## Footnotes

1 Modern test theory includes frameworks like Item Response Theory and Rasch Analysis, which involve modelling the relationship between an individual’s latent trait and the pattern of test item responses, rather than focussing on the test score, as characterises CTT.
