# Supplementary material for: The Multimorbidity Questionnaire (MMQ1): English translation and validation of a Danish patient reported outcome measure for quality of life in people with multiple long-term conditions in a cross-sectional survey
Source: Qual Life Res. 2025 Jan 23;34(5):1291–304. doi: 10.1007/s11136-025-03901-6 (PMC12064619; doi:10.1007/s11136-025-03901-6)
Supplement: Supplementary file 1 — Supplementary file1 (DOCX 1160 kb) [file 11136_2025_3901_MOESM1_ESM.docx]

**Supplementary material**

Supplementary Figure 1. Distribution of responses


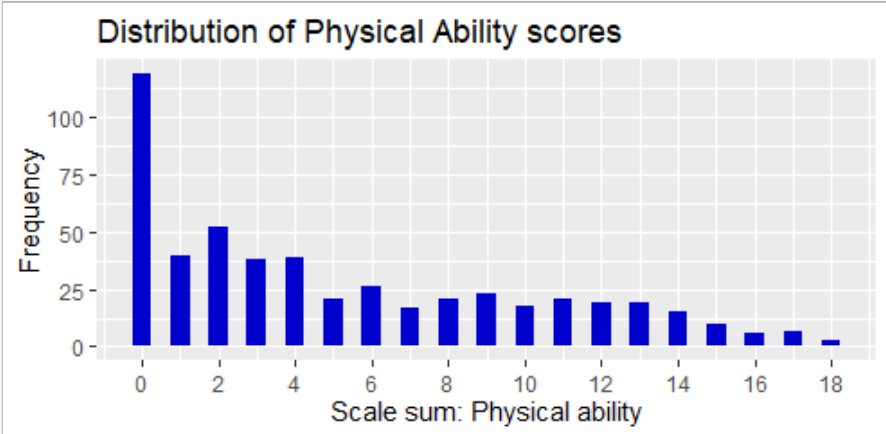

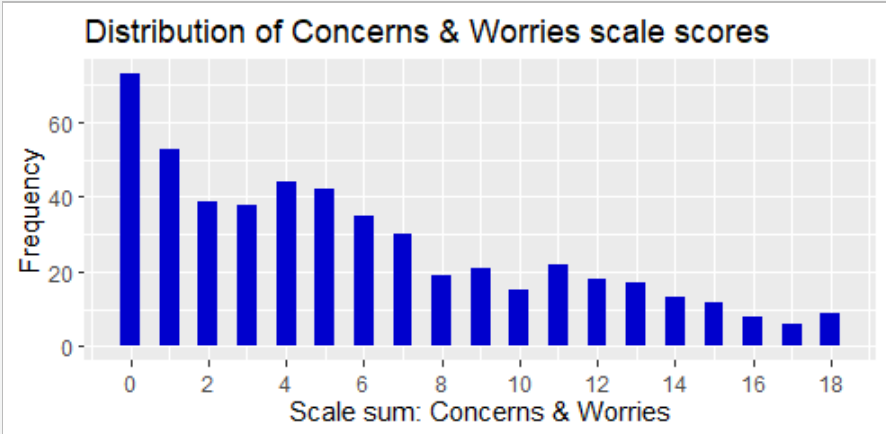

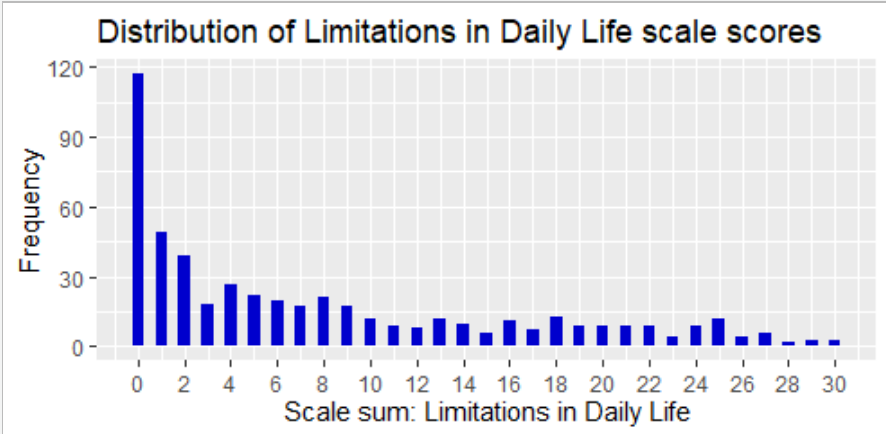

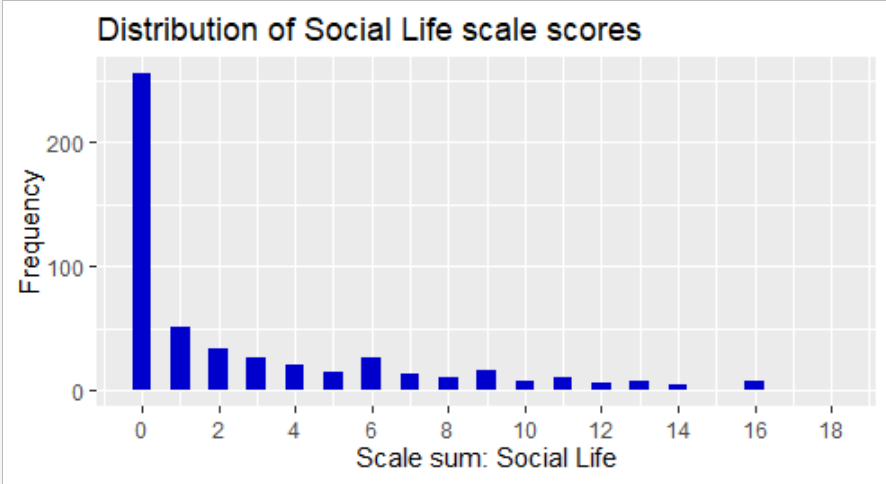

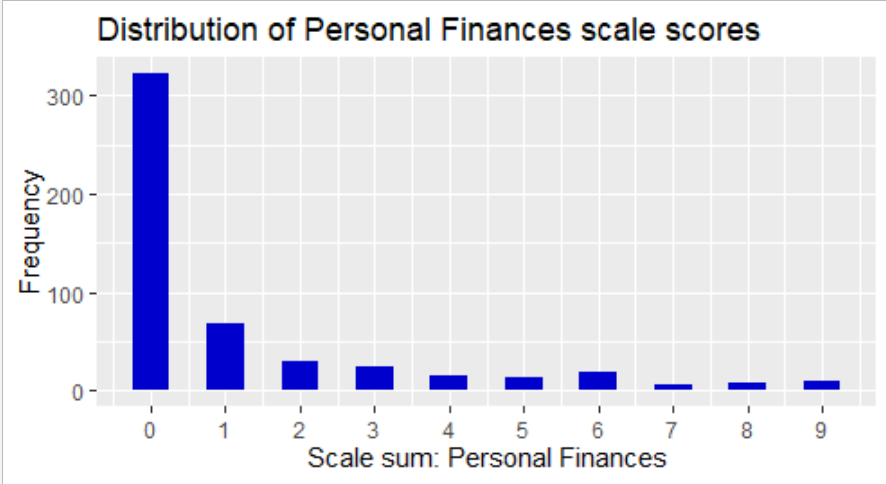

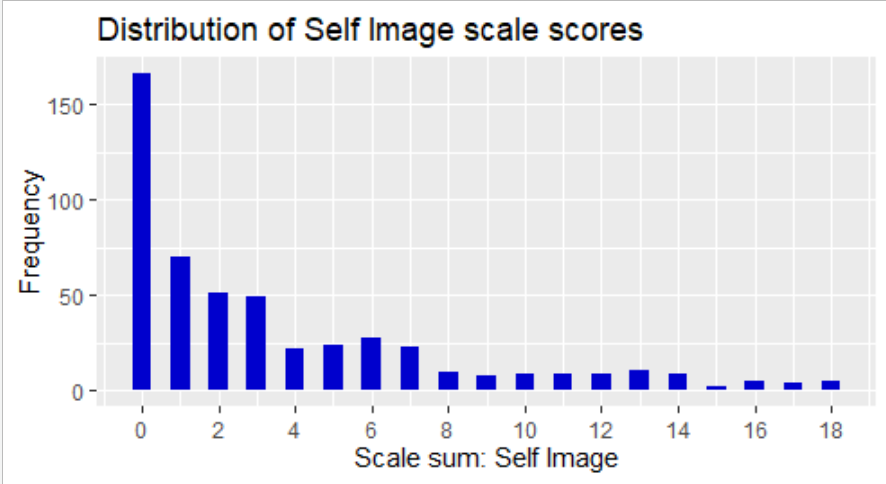


Supplementary Table 1. Inter-item Correlation Matrices

| Scale 1 | q1a | q1b | q1c | q1d | q1e | q1f |  |  |  |  |
| --- | --- | --- | --- | --- | --- | --- | --- | --- | --- | --- |
| q1a | 1 | 0.775 | 0.545 | 0.710 | 0.743 | 0.764 |  |  |  |  |
| q1b | 0.775 | 1 | 0.522 | 0.726 | 0.726 | 0.793 |  |  |  |  |
| q1c | 0.545 | 0.522 | 1 | 0.632 | 0.529 | 0.483 |  |  |  |  |
| q1d | 0.710 | 0.726 | 0.632 | 1 | 0.732 | 0.724 |  |  |  |  |
| q1e | 0.743 | 0.726 | 0.529 | 0.732 | 1 | 0.809 |  |  |  |  |
| q1f | 0.764 | 0.793 | 0.483 | 0.724 | 0.809 | 1 |  |  |  |  |
|  | | | | | | |  |  |  |  |
| Scale 2 | q2a | q2b | q2c | q2d | q2e | q2f |  |  |  |  |
| q2a | 1 | 0.604 | 0.787 | 0.716 | 0.659 | 0.657 |  |  |  |  |
| q2b | 0.604 | 1 | 0.659 | 0.639 | 0.524 | 0.517 |  |  |  |  |
| q2c | 0.787 | 0.659 | 1 | 0.751 | 0.657 | 0.633 |  |  |  |  |
| q2d | 0.716 | 0.639 | 0.751 | 1 | 0.670 | 0.686 |  |  |  |  |
| q2e | 0.659 | 0.524 | 0.657 | 0.670 | 1 | 0.657 |  |  |  |  |
| q2f | 0.657 | 0.517 | 0.633 | 0.686 | 0.657 | 1 |  |  |  |  |
|  | | | | | | |  |  |  |  |
| Scale 3 | q3a | q3b | q3c | q3d | q3e | q3f | q3g | q3h | q3i | q3j |
| q3a | 1 | 0.828 | 0.757 | 0.805 | 0.745 | 0.742 | 0.762 | 0.575 | 0.664 | 0.764 |
| q3b | 0.828 | 1 | 0.785 | 0.802 | 0.821 | 0.784 | 0.78 | 0.617 | 0.697 | 0.762 |
| q3c | 0.757 | 0.785 | 1 | 0.716 | 0.752 | 0.746 | 0.789 | 0.688 | 0.721 | 0.681 |
| q3d | 0.805 | 0.802 | 0.716 | 1 | 0.743 | 0.725 | 0.730 | 0.590 | 0.692 | 0.787 |
| q3e | 0.745 | 0.821 | 0.752 | 0.743 | 1 | 0.781 | 0.723 | 0.571 | 0.634 | 0.670 |
| q3f | 0.742 | 0.784 | 0.746 | 0.725 | 0.781 | 1 | 0.723 | 0.612 | 0.689 | 0.707 |
| q3g | 0.762 | 0.780 | 0.789 | 0.730 | 0.723 | 0.723 | 1 | 0.656 | 0.713 | 0.684 |
| q3h | 0.575 | 0.617 | 0.688 | 0.590 | 0.571 | 0.612 | 0.656 | 1 | 0.612 | 0.563 |
| q3i | 0.664 | 0.697 | 0.721 | 0.692 | 0.634 | 0.689 | 0.713 | 0.612 | 1 | 0.696 |
| q3j | 0.764 | 0.762 | 0.681 | 0.787 | 0.670 | 0.707 | 0.684 | 0.563 | 0.696 | 1 |
|  | | | | | | | | | | |
| Scale 4 | q4a | q4b | q4c | q4d | q4e | q4f |  |  |  |  |
| q4a | 1 | 0.723 | 0.571 | 0.631 | 0.748 | 0.643 |  |  |  |  |
| q4b | 0.723 | 1 | 0.581 | 0.653 | 0.666 | 0.690 |  |  |  |  |
| q4c | 0.571 | 0.581 | 1 | 0.587 | 0.526 | 0.583 |  |  |  |  |
| q4d | 0.631 | 0.653 | 0.587 | 1 | 0.607 | 0.595 |  |  |  |  |
| q4e | 0.748 | 0.666 | 0.526 | 0.607 | 1 | 0.652 |  |  |  |  |
| q4f | 0.643 | 0.690 | 0.583 | 0.595 | 0.652 | 1 |  |  |  |  |
|  |  |  |  |  |  |  |  |  |  |  |
| Scale 5 | q5a | q5b | q5c |  |  |  |  |  |  |  |
| q5a | 1 | 0.797 | 0.777 |  |  |  |  |  |  |  |
| q5b | 0.797 | 1 | 0.752 |  |  |  |  |  |  |  |
| q5c | 0.777 | 0.752 | 1 |  |  |  |  |  |  |  |
|  |  |  |  |  |  |  |  |  |  |  |
| Scale 6 | q6a | q6b | q6c | q6d | q6e | q6f |  |  |  |  |
| q6a | 1 | 0.831 | 0.731 | 0.678 | 0.502 | 0.424 |  |  |  |  |
| q6b | 0.831 | 1 | 0.740 | 0.679 | 0.537 | 0.468 |  |  |  |  |
| q6c | 0.731 | 0.740 | 1 | 0.714 | 0.454 | 0.392 |  |  |  |  |
| q6d | 0.678 | 0.679 | 0.714 | 1 | 0.469 | 0.384 |  |  |  |  |
| q6e | 0.502 | 0.537 | 0.454 | 0.469 | 1 | 0.682 |  |  |  |  |
| q6f | 0.424 | 0.468 | 0.392 | 0.384 | 0.682 | 1 |  |  |  |  |

Supplementary Figure 3. Illustrative quotes from cognitive interviews

Importance and relevance

*You can go from someone like me, with few restrictions, to someone who is totally reliant on help - and the questions stay relevant. I would say it’s totally valid for, probably, 90 per cent of older folk. There will be people around here that could write a book on some of this stuff. (P5)*

*You need to ask this because poverty is probably one of the major contributing factors to peoples’ state of mind. (P5)*

*The questions are straight to the point. Am I a burden to others? I think that’s quite a good one in particular. (P4)*

*Do I feel guilty in relation to my lifestyle? It’s a good question but it’s a tough one to answer – I do feel guilty but also I feel proud to have stopped drinking. (P6)*

Clarity and comprehensibility

*That was pretty straightforward to understand. I felt comfortable with the wording. (P1)*

*I didn’t really have any issues understanding the questions. Each of them made me think a little bit, so they’re there for a reason. (P2)*

*I feel the questions are clear, the way they’re phrased and the way they’re laid out. It’s very easy to follow. (P4)*

Coverage

*That section [MMQ1] seems bang on in terms of what it covers. (P2)*

*You are certainly asking the right questions. I think the whole study – these questions – are really good. Really worthwhile. Not only now but in the future with ageing getting longer. (P4)*

*To me it felt like one or two of these questions are the ultimate questions. But then the others are giving more reasons why somebody feels that way. So I think you’ve got to ask them all. (P3)*

Burden

*Q: If you were given this as a research participant would you have any issues with it?
A: It’s too big an assessment. (P1)*

*Maybe there could be fewer questions. I personally think so. Some of them don’t need to be there. It’s like when you’re doing an online course at work and it takes, like, two years to complete. You can shorten it, surely. (P2)*

*Q: Did you feel like it was a burdensome thing to fill out?*

*A: Not really. In certain sections it was repetitive, but for other people those sections might be important. (P5)*

Supplementary Table 2. Concurrent validity with EQ-5D-5L and ICE-CAP

Correlation (Spearman coefficients) between MMQ1 scale scores and comparator measures

|  |  | **ICE-CAP** | | | | | |
| --- | --- | --- | --- | --- | --- | --- | --- |
|  |  | Item A | Item B | Item C | Item D | Item E | Total |
| **MMQ** | Scale1 | 0.595 | 0.345 | 0.724 | 0.707 | 0.618 | 0.756 |
|  | Scale 2 | 0.666 | 0.383 | 0.607 | 0.632 | 0.626 | 0.731 |
|  | Scale 3 | 0.603 | 0.349 | 0.726 | 0.707 | 0.634 | 0.760 |
|  | Scale4 | 0.613 | 0.420 | 0.705 | 0.689 | 0.668 | 0.767 |
|  | Scale5 | 0.510 | 0.361 | 0.450 | 0.465 | 0.513 | 0.559 |
|  | Scale6 | 0.583 | 0.421 | 0.556 | 0.579 | 0.595 | 0.673 |
|  | Total | 0.677 | 0.413 | 0.728 | 0.729 | 0.682 | 0.813 |
|  | | | | | | | |
|  |  | **EQ-5D-5L** | | | | | |
|  |  | Item A | Item B | Item C | Item D | Item E | Total |
| **MMQ** | Scale 1 | 0.758 | 0.652 | 0.820 | 0.635 | 0.482 | 0.847 |
|  | Scale 2 | 0.585 | 0.530 | 0.706 | 0.592 | 0.623 | 0.765 |
|  | Scale 3 | 0.735 | 0.645 | 0.817 | 0.616 | 0.509 | 0.842 |
|  | Scale 4 | 0.633 | 0.644 | 0.724 | 0.527 | 0.557 | 0.756 |
|  | Scale 5 | 0.367 | 0.457 | 0.486 | 0.415 | 0.440 | 0.520 |
|  | Scale 6 | 0.504 | 0.504 | 0.612 | 0.493 | 0.590 | 0.666 |
|  | Total | 0.710 | 0.638 | 0.818 | 0.631 | 0.600 | 0.860 |

|  |  | Single-item QOL rating |
| --- | --- | --- |
| **MMQ** | Scale1 | 0.711 |
|  | Scale 2 | 0.717 |
|  | Scale 3 | 0.727 |
|  | Scale4 | 0.718 |
|  | Scale5 | 0.507 |
|  | Scale6 | 0.662 |
|  | Total | 0.771 |

Supplementary Table 3. Convergent and discriminant validity

Item-total correlations (Spearman coefficients) between scale items and scale sum scores, showing that for all items, correlation is highest with the native scale.

|  | Items from Scale 1 | | | | | | | | | |  |  | | |  |  | | |  |  |  |  |
| --- | --- | --- | --- | --- | --- | --- | --- | --- | --- | --- | --- | --- | --- | --- | --- | --- | --- | --- | --- | --- | --- | --- |
|  | Item A | | Item B | | Item C | | | Item D | Item E | Item F |  |  | | |  |  | | |  |  |  |  |
| **Scale 1 sum** | 0.831 | | 0.834 | | 0.602 | | | 0.817 | 0.833 | 0.848 |  |  | | |  |  | | |  |  |  |  |
| Scale 2 sum | 0.686 | | 0.652 | | 0.469 | | | 0.612 | 0.745 | 0.740 |  |  | | |  |  | | |  |  |  |  |
| Scale 3 sum | 0.787 | | 0.789 | | 0.563 | | | 0.767 | 0.803 | 0.861 |  |  | | |  |  | | |  |  |  |  |
| Scale 4 sum | 0.721 | | 0.699 | | 0.576 | | | 0.727 | 0.705 | 0.732 |  |  | | |  |  | | |  |  |  |  |
| Scale 5 sum | 0.474 | | 0.432 | | 0.454 | | | 0.474 | 0.454 | 0.430 |  |  | | |  |  | | |  |  |  |  |
| Scale 6 sum | 0.622 | | 0.583 | | 0.451 | | | 0.560 | 0.640 | 0.650 |  |  | | |  |  | | |  |  |  |  |
|  |  |  | |  | |  |  |  |  |  |  |  |  |  |  |  |  |  |  |  |  |  |
|  | Items from Scale 2 | | | | | | | | | |  | |  | | | |  | | |  | |  |
|  | Item A | | Item B | | Item C | | | Item D | Item E | Item F |  | |  | | | |  | | |  | |  |
| Scale 1 sum | 0.615 | | 0.464 | | 0.587 | | | 0.807 | 0.642 | 0.676 |  | |  | | | |  | | |  | |  |
| **Scale 2 sum** | 0.812 | | 0.678 | | 0.827 | | | 0.823 | 0.743 | 0.738 |  | |  | | | |  | | |  | |  |
| Scale 3 sum | 0.637 | | 0.498 | | 0.616 | | | 0.782 | 0.664 | 0.725 |  | |  | | | |  | | |  | |  |
| Scale 4 sum | 0.629 | | 0.484 | | 0.596 | | | 0.696 | 0.663 | 0.707 |  | |  | | | |  | | |  | |  |
| Scale 5 sum | 0.492 | | 0.393 | | 0.466 | | | 0.488 | 0.470 | 0.468 |  | |  | | | |  | | |  | |  |
| Scale 6 sum | 0.659 | | 0.538 | | 0.642 | | | 0.678 | 0.676 | 0.649 |  | |  | | | |  | | |  | |  |
|  |  | |  | |  | | |  |  |  |  |  |  |  |  |  |  |  |  |  |  |  |
|  | Items from Scale 3 | | | | | | | | | | | | | | | | | | | | |  |
|  | Item A | | Item B | | Item C | | | Item D | Item E | Item F | Item G | | Item H | | | | Item I | | | Item J | |  |
| Scale 1 sum | 0.798 | | 0.809 | | 0.719 | | | 0.836 | 0.758 | 0.747 | 0.757 | | 0.579 | | | | 0.715 | | | 0.789 | |  |
| Scale 2 sum | 0.698 | | 0.746 | | 0.617 | | | 0.725 | 0.680 | 0.687 | 0.651 | | 0.569 | | | | 0.635 | | | 0.674 | |  |
| **Scale 3 sum** | 0.862 | | 0.895 | | 0.855 | | | 0.854 | 0.831 | 0.840 | 0.845 | | 0.694 | | | | 0.783 | | | 0.813 | |  |
| Scale 4 sum | 0.754 | | 0.800 | | 0.769 | | | 0.759 | 0.765 | 0.764 | 0.777 | | 0.629 | | | | 0.741 | | | 0.703 | |  |
| Scale 5 sum | 0.404 | | 0.485 | | 0.494 | | | 0.449 | 0.471 | 0.474 | 0.469 | | 0.462 | | | | 0.534 | | | 0.472 | |  |
| Scale 6 sum | 0.623 | | 0.662 | | 0.570 | | | 0.654 | 0.637 | 0.616 | 0.567 | | 0.537 | | | | 0.616 | | | 0.620 | |  |
|  |  |  |  |  |  |  |  |  |  |  |  |  |  |  |  |  |  |  |  |  |  |  |
|  | Items from Scale 4 | | | | | | | | | |  | | |  | | | |  | | |  | |
|  | Item A | | Item B | | Item C | | | Item D | Item E | Item F |  | | |  | | | |  | | |  | |
| Scale 1 sum | 0.698 | | 0.716 | | 0.450 | | | 0.580 | 0.635 | 0.601 |  | | |  | | | |  | | |  | |
| Scale 2 sum | 0.630 | | 0.702 | | 0.506 | | | 0.532 | 0.601 | 0.568 |  | | |  | | | |  | | |  | |
| Scale 3 sum | 0.752 | | 0.770 | | 0.492 | | | 0.613 | 0.686 | 0.639 |  | | |  | | | |  | | |  | |
| **Scale 4 sum** | 0.804 | | 0.798 | | 0.664 | | | 0.727 | 0.770 | 0.755 |  | | |  | | | |  | | |  | |
| Scale 5 sum | 0.472 | | 0.505 | | 0.425 | | | 0.492 | 0.507 | 0.498 |  | | |  | | | |  | | |  | |
| Scale 6 sum | 0.610 | | 0.661 | | 0.479 | | | 0.550 | 0.641 | 0.549 |  | | |  | | | |  | | |  | |
|  |  | |  | |  | | |  |  |  |  | | |  | | | |  | | |  | |
|  | Items from Scale 5 | | | | | | |  |  |  |  | | |  | | | |  | | |  | |
|  | Item A | | Item B | | Item C | | |  |  |  |  | | |  | | | |  | | |  | |
| Scale 1 sum | 0.509 | | 0.449 | | 0.426 | | |  |  |  |  | | |  | | | |  | | |  | |
| Scale 2 sum | 0.536 | | 0.503 | | 0.460 | | |  |  |  |  | | |  | | | |  | | |  | |
| Scale 3 sum | 0.553 | | 0.451 | | 0.438 | | |  |  |  |  | | |  | | | |  | | |  | |
| Scale 4 sum | 0.580 | | 0.489 | | 0.481 | | |  |  |  |  | | |  | | | |  | | |  | |
| **Scale 5 sum** | 0.841 | | 0.823 | | 0.806 | | |  |  |  |  | | |  | | | |  | | |  | |
| Scale 6 sum | 0.527 | | 0.490 | | 0.466 | | |  |  |  |  | | |  | | | |  | | |  | |
|  |  | |  | |  | | |  |  |  |  | | |  | | | |  | | |  | |
|  | Items from Scale 6 | | | | | | | | | |  | | |  | | | |  | | |  | |
|  | Item A | | Item B | | Item C | | | Item D | Item E | Item F |  | | |  | | | |  | | |  | |
| Scale 1 sum | 0.677 | | 0.666 | | 0.537 | | | 0.539 | 0.373 | 0.308 |  | | |  | | | |  | | |  | |
| Scale 2 sum | 0.693 | | 0.747 | | 0.585 | | | 0.568 | 0.506 | 0.409 |  | | |  | | | |  | | |  | |
| Scale 3 sum | 0.735 | | 0.709 | | 0.562 | | | 0.587 | 0.410 | 0.303 |  | | |  | | | |  | | |  | |
| Scale 4 sum | 0.740 | | 0.742 | | 0.605 | | | 0.625 | 0.452 | 0.364 |  | | |  | | | |  | | |  | |
| Scale 5 sum | 0.542 | | 0.504 | | 0.506 | | | 0.506 | 0.343 | 0.290 |  | | |  | | | |  | | |  | |
| **Scale 6 sum** | 0.795 | | 0.820 | | 0.753 | | | 0.722 | 0.636 | 0.560 |  | | |  | | | |  | | |  | |

Supplementary Table 4. Comparison of factor loadings for two CFA models used for scale six (Self-image)

| Item and content | | Model 1  Factor loadings (SE) | Model 2*  Factor loadings (SE) |
| --- | --- | --- | --- |
|  | |  |  |
| 6a | I feel embarrassed about the impact and limitations my health conditions cause | 0.935 (0.011) | 0.939 (0.011) |
| 6b | My health conditions lower my self-esteem | 0.947 (0.010) | 0.956 (0.010) |
| 6c | It is unpleasant being seen as a patient by others | 0.886 (0.015) | 0.897 (0.015) |
| 6d | I feel people judge me because of my health conditions | 0.854 (0.018) | 0.868 (0.018) |
| 6e | I blame myself for my health conditions | 0.802 (0.024) | 0.657 (0.035) |
| 6f | I often feel guilty about my lifestyle (e.g. smoking, alcohol, diet, exercise) in relation to my health conditions | 0.754 (0.026) | 0.558 (0.040) |
| *Model 2 includes correlation term: 6e ~~ 6f | | | |

Supplementary Table 5. Inter-scale correlation

Correlation (Spearman coefficients) between sum scores for each of the six scales in MMQ1

|  | sum_q1 | sum_q2 | sum_q3 | sum_q4 | sum_q5 | sum_q6 |
| --- | --- | --- | --- | --- | --- | --- |
| sum_q1 | 1.000 | 0.767 | 0.898 | 0.787 | 0.502 | 0.674 |
| sum_q2 | 0.767 | 1.000 | 0.789 | 0.748 | 0.543 | 0.765 |
| sum_q3 | 0.898 | 0.789 | 1.000 | 0.848 | 0.517 | 0.712 |
| sum_q4 | 0.787 | 0.748 | 0.848 | 1.000 | 0.555 | 0.736 |
| sum_q5 | 0.502 | 0.543 | 0.517 | 0.555 | 1.000 | 0.532 |
| sum_q6 | 0.674 | 0.765 | 0.712 | 0.736 | 0.532 | 1.000 |

Sampling strategy

**Cognitive interview sampling**

Criteria: Adult patients living with MLTC, where the GP felt the health conditions were likely to be impacting on quality of life.

Sampling aimed for a balance of genders, and with good representation of those from more deprived areas and/or with mental-physical multimorbidity. Sampling was facilitated by two GP practices, one in an area of mixed deprivation, the other in an area of high deprivation. Potentially suitable patients were identified by their GP, either opportunistically or from clinical familiarity, and contacted by their GP by telephone.

**9** patients were approached by their GP and consented to be contacted by the research team

**6** patient responded to contact and agreed to interview

**Survey sampling**

Criteria: Adult patients living with MLTC, who would be able to complete a short paper-based survey.

Search strategy on practice records: Patients on two or more chronic disease registers, or four or more repeat prescriptions, who had not been recently invited to another research study.

Chronic disease registers included: coronary heart disease, heart failure, stroke, diabetes, severe mental illness, dementia, chronic kidney disease, atrial fibrillation, peripheral arterial disease, chronic obstructive pulmonary disease, asthma, rheumatoid arthritis, hypertension, cancer.

Eight practice supported sampling. These practices were recruited by volunteering in response to an advert circulated by NHS Research Scotland Primary Care Network.

A sample list for screening was identified randomly from all patients meeting the search criteria on practice records. The size of the screening list was either 200 or 400 based on participation GP discretion. Patients deemed unsuitable for inclusion by their GP (e.g., learning disability, dementia, recent distressing life event, approaching end-of-life).

**11,860** potentially eligible patients identified based on search of 8 practice lists

**2,800** patients randomly sampled from those eligible

**47** deemed unsuitable

**2753** retained for invitation after screening by GP for suitability

**597** responses received at closure of data collection

Survey changes following cognitive interviews

The following changes (in *underlined* *italics*) were made to the wording of MMQ1 following first round of cognitive interviews

1. Item 2e

“I *have to* make an effort to stay mentally strong”

1. Item 6f

“I often feel guilty about my lifestyle (*e.g. smoking, alcohol, diet, exercise*) in relation to my health conditions”
